# Supplementary material for: Relationships among COVID-19 phobia, health anxiety, and social relations in women living with HIV in Iran: A path analysis
Source: PLoS One. 2022 Oct 4;17(10):e0275455. doi: 10.1371/journal.pone.0275455 (PMC9531807; doi:10.1371/journal.pone.0275455)
Supplement: Attachment — Submitted filename: PONE-D-22-08845_Reviewer Comments.pdf [file pone.0275455.s002.pdf]

PLOS ONE

Relationships among Corona phobia, health anxiety, and social relations in people living with HIV (PLHIV): A path analysis

--Manuscript Draft--

**Manuscript Number:**	PONE-D-22-08845
**Article Type:**	Research Article
**Full Title:**	Relationships among Corona phobia, health anxiety, and social relations in people living with HIV (PLHIV): A path analysis
**Short Title:**	Corona phobia, anxiety, and social relations in people living with HIV
**Corresponding Author:**	Mostafa Qorbani, PhD<br>School of Medicine, Alborz University of Medical Sciences<br>Tehran, IRAN, ISLAMIC REPUBLIC OF
**Keywords:**	Health anxiety; COVID-19; Corona phobia; Social relations; Path analysis
**Abstract:**	Background<br><br>The COVID-19 pandemic and its consequences have caused fear and anxiety worldwide. This study aimed to determine the relationships among Corona phobia, health anxiety, and social relations in people living with HIV (PLHIV).<br><br>Methods<br><br>This descriptive study enrolled 300 PLHIV who had records at the Behavioral Diseases Center of Tehran University of Medical Sciences. The data were collected via three questionnaires: the Fear of COVID-19 Scale, social relations questionnaire, and sociodemographic information checklist, and analyzed in SPSS 25 and LISREL 8.8.<br><br>Results<br><br>Based on the path analysis, among variables that had significant causal relationships with social relations, socioeconomic status (B = -0.14) showed the greatest negative relationship, and health anxiety (B = 0.11) had the greatest positive relationship on the direct path. However, fear of COVID-19 (B = 0.049) displayed the greatest positive relationship on the indirect path. The level of education (B = 0.29) was the only variable showing a significant and positive relationship with social relations on both direct and indirect paths.<br><br>Conclusion<br><br>This study was conducted during the COVID-19 pandemic when it was necessary to adhere to social distancing and limiting social relations. A better socioeconomic status was related to fewer social relations. Moreover, increased fear and health anxiety related to a higher social relations score. Due to their vulnerability, PLHIV require more support and education to adhere to health protocols.
**Order of Authors:**	Fatemeh Aliverdi
	Zahra Bayat Jozani
	Nooshin Ghavidel
	Mostafa Qorbani, PhD
	Nami Mohammadian Khonsari
	Farima Mohamadi
	Minoo Mohraz
	Zohreh Mahmoodi
**Additional Information:**	

Powered by Editorial Manager® and ProduXion Manager® from Aries Systems Corporation

Question	Response
**Financial Disclosure**<br><br>Enter a financial disclosure statement that describes the sources of funding for the work included in this submission. Review the submission guidelines for detailed requirements. View published research articles from *PLOS ONE* for specific examples.<br><br>This statement is required for submission and **will appear in the published article** if the submission is accepted. Please make sure it is accurate.<br><br>**Unfunded studies**<br>Enter: *The author(s) received no specific funding for this work.*<br><br>**Funded studies**<br>Enter a statement with the following details:<br>• Initials of the authors who received each award<br>• Grant numbers awarded to each author<br>• The full name of each funder<br>• URL of each funder website<br>• Did the sponsors or funders play any role in the study design, data collection and analysis, decision to publish, or preparation of the manuscript?<br>• **NO** - Include this sentence at the end of your statement: *The funders had no role in study design, data collection and analysis, decision to publish, or preparation of the manuscript.*<br>• **YES** - Specify the role(s) played.<br><br>* typeset	The authors received no specific funding for this work.<br>This research did not receive any specific grant from funding agencies in the public, commercial, or not-for-profit sectors.
**Competing Interests**<br><br>Use the instructions below to enter a competing interest statement for this submission. On behalf of all authors, disclose any competing interests that could be perceived to bias this work—acknowledging all financial support and any other relevant financial or non-financial competing interests.	The authors declare that they have no competing interests

This statement is required for submission and **will appear in the published article** if the submission is accepted. Please make sure it is accurate and that any funding sources listed in your Funding Information later in the submission form are also declared in your Financial Disclosure statement.<br><br>View published research articles from *PLOS ONE* for specific examples.<br><br>**NO authors have competing interests**<br><br>Enter: *The authors have declared that no competing interests exist.*<br><br>**Authors with competing interests**<br><br>Enter competing interest details beginning with this statement:<br><br>*I have read the journal's policy and the authors of this manuscript have the following competing interests: [insert competing interests here]*<br><br>* typeset	
**Ethics Statement**<br><br>Enter an ethics statement for this submission. This statement is required if the study involved:<br><br>• Human participants<br>• Human specimens or tissue<br>• Vertebrate animals or cephalopods<br>• Vertebrate embryos or tissues<br>• Field research<br><br>Write "N/A" if the submission does not require an ethics statement.<br><br>General guidance is provided below. Consult the submission guidelines for detailed instructions. **Make sure that all information entered here is included in the Methods section of the manuscript.**	Informed consent was obtained from all the participants entering the study. Relevant guidelines and regulations were observed for all methods. All experimental protocols were approved by the Ethics Committee of Alborz University of Medical Sciences (Abzums.Rec.1399.234). All methods were carried out in accordance with relevant guidelines and regulations

**Format for specific study types**<br><br>**Human Subject Research (involving human participants and/or tissue)**<br>• Give the name of the institutional review board or ethics committee that approved the study<br>• Include the approval number and/or a statement indicating approval of this research<br>• Indicate the form of consent obtained (written/oral) or the reason that consent was not obtained (e.g. the data were analyzed anonymously)<br><br>**Animal Research (involving vertebrate animals, embryos or tissues)**<br>• Provide the name of the Institutional Animal Care and Use Committee (IACUC) or other relevant ethics board that reviewed the study protocol, and indicate whether they approved this research or granted a formal waiver of ethical approval<br>• Include an approval number if one was obtained<br>• If the study involved *non-human primates*, add *additional details* about animal welfare and steps taken to ameliorate suffering<br>• If anesthesia, euthanasia, or any kind of animal sacrifice is part of the study, include briefly which substances and/or methods were applied<br><br>**Field Research**<br><br>Include the following details if this study involves the collection of plant, animal, or other materials from a natural setting:<br>• Field permit number<br>• Name of the institution or relevant body that granted permission	
**Data Availability**<br><br>Authors are required to make all data underlying the findings described fully available, without restriction, and from the time of publication. PLOS allows rare exceptions to address legal and ethical concerns. See the PLOS Data Policy and FAQ for detailed information.	No - some restrictions will apply

Powered by Editorial Manager® and ProduXion Manager® from Aries Systems Corporation

A Data Availability Statement describing where the data can be found is required at submission. Your answers to this question constitute the Data Availability Statement and **will be published in the article**, if accepted.<br><br>**Important:** Stating 'data available on request from the author' is not sufficient. If your data are only available upon request, select 'No' for the first question and explain your exceptional situation in the text box.<br><br>Do the authors confirm that all data underlying the findings described in their manuscript are fully available without restriction?	
**Describe where the data may be found in full sentences. If you are copying our sample text, replace any instances of XXX with the appropriate details.**<br><br>• If the data are **held or will be held in a public repository**, include URLs, accession numbers or DOIs. If this information will only be available after acceptance, indicate this by ticking the box below. For example: *All XXX files are available from the XXX database (accession number(s) XXX, XXX.).*<br>• If the data are all contained **within the manuscript and/or Supporting Information files**, enter the following: *All relevant data are within the manuscript and its Supporting Information files.*<br>• If neither of these applies but you are able to provide **details of access elsewhere**, with or without limitations, please do so. For example:<br><br>*Data cannot be shared publicly because of [XXX]. Data are available from the XXX Institutional Data Access / Ethics Committee (contact via XXX) for researchers who meet the criteria for access to confidential data.*<br><br>*The data underlying the results presented in the study are available*	The data that support the findings of this study are available from the corresponding author upon reasonable request

from (include the name of the third party and contact information or URL).<br>• This text is appropriate if the data are owned by a third party and authors do not have permission to share the data.<br><br>* typeset	
Additional data availability information:	

Powered by Editorial Manager® and ProduXion Manager® from Aries Systems Corporation

Relationships among Corona phobia, health anxiety, and social relations in people living with HIV (PLHIV): A path analysis

Fatemeh Aliverdi [1], Zahra Bayat Jozani[2] , Nooshin Ghavidel [3] , Mostafa Qorbani[4a,4b*] , Nami Mohammadian Khonsari[4a] ,Farima Mohamadi[5] , Minoo Mohraz[6] ,Zohreh Mahmoodi[7]*

[1-] Student Research Committee ,Alborz University of Medical Sciences, Karaj, Iran

2-PhD student .Department of Reproductive Health and Midwifery, School of Nursing and Midwifery, Tehran University of Medical Sciences ,Tehran, IRAN

3- Social Determinants of Health Research Center, Alborz University of Medical Sciences, Karaj, Iran

4-a: Non-Communicable Diseases Research Center, Alborz University of Medical Sciences, Karaj, Iran 4-b: Endocrinology and Metabolism Research Center, Endocrinology and Metabolism Clinical Sciences Institute, Tehran University of Medical Sciences, Tehran, Iran

5- Social Determinants of Health Research Center, shahid beheshti University of Medical Sciences, Tehran, Iran

6- Iranian Research Center for HIV/AIDS, Iranian Institute for Reduction of High Risk Behaviors, Tehran University of Medical Sciences, Tehran, Iran

7- Social Determinants of Health Research Center, Alborz University of Medical Sciences, Karaj, Iran

*Equally contributed as corresponding authors: Zohreh Mahmoodi: Social Determinants of Health Research Center, Alborz University of Medical Sciences, Karaj, Iran

Email:Zohrehmahmoodi2011@gmail.com

Mostafa Qorbani: Non-Communicable Diseases Research Center, Alborz University of Medical Sciences

Email: mqorbani1379@yahoo.com

Abstract:

Background: The COVID-19 pandemic and its consequences have caused fear and anxiety worldwide. This study aimed to determine the relationships among Corona phobia, health anxiety, and social relations in people living with HIV (PLHIV).

Methods: This descriptive study enrolled 300 PLHIV who had records at the Behavioral Diseases Center of Tehran University of Medical Sciences. The data were collected via three questionnaires: the Fear of COVID-19 Scale, social relations questionnaire, and sociodemographic information checklist, and analyzed in SPSS 25 and LISREL 8.8.

Results: Based on the path analysis, among variables that had significant causal relationships with social relations, socioeconomic status (B = -0.14) showed the greatest negative relationship, and health anxiety (B = 0.11) had the greatest positive relationship on the direct path. However, fear of COVID-19 (B = 0.049) displayed the greatest positive relationship on the indirect path. The level of education (B = 0.29) was the only variable showing a significant and positive relationship with social relations on both direct and indirect paths.

Conclusion: This study was conducted during the COVID-19 pandemic when it was necessary to adhere to social distancing and limiting social relations. A better socioeconomic status was related to fewer social relations. Moreover, increased fear and health anxiety related to a higher social relations score. Due to their vulnerability, PLHIV require more support and education to adhere to health protocols.

Keywords: Health anxiety, COVID-19, Corona phobia, Social relations, Path analysis

1. Background:

The COVID-19 pandemic has affected people’s psychological status (Fofana, Latif, Sarfraz, Bashir, & Komal). Based on the Inter-Agency Standing Committee (IASC) report, people are, directly and indirectly, impacted by stressful experiences in this period. The most prevalent responses include fear (of illness, death, loss of livelihood, social isolation, and being quarantined)

and fear-related behaviours, e.g., limited social relations, distance from treatment centers, health anxiety, depression, and stress (Porcelli)

Fear is an adaptive feeling needed to cope with potential threats, but excessive fear has negative impacts at the personal (mental health issues and anxiety disorders) and social level (seclusion, isolation, xenophobia) (Mertens, Gerritsen, Duijndam, Salemink, & Engelhard). Researchers have discussed the pathological fear of COVID-19 (corona phobia) due to the nature and wide-ranging impacts of the pandemic (Asmundson & Taylor)

Various factors may affect the degree of psychological vulnerability to corona phobia, including personal variables such as tolerance, lack of trust, vulnerability to the diseases, anxiety, and concerns (Asmundson & Taylor). Reports suggest that older adults and those with underlying diseases, including HIV, run a greater risk (Control & Prevention). It is expected that the COVID-19 pandemic imposes a more significant physical and psychological burden on people living with HIV (PLHIV) (Chenneville, Gabbidon, Hanson, & Holyfield, 2020; Tunçel et al., 2020). This group lives with severe early death anxiety, different types of fears related to this disease, mental disturbances ranging from indifference and hopelessness to severe reactions such as anxiety and depressive disorders (Belir, Ansari Shahidi, & Mohammadi).

Health anxiety is a wide-ranging cognitive disorder formed as incorrect perceptions about physical changes and symptoms resulting from one’s beliefs about illness or health (Solem et al.). According to some researchers, health anxiety is a major psychological factor related to corona phobia (Asmundson & Taylor). Almost everyone has experienced some degrees of health anxiety, low levels of which are not pathological but rather help people perform and commit to preventive behaviours. However, its severe degrees are associated with maladaptive coping behaviours leading to distress, social incompetence, disrupted job performance, and repeated visits to health centers (Salkovskis & Warwick).

People inherently need and thus create opportunities to experience social relations. Social relations also affect mental health, health-related behaviours, and physical health. Studies show that social relations have short- and long-term effects on health that emerge during childhood and throughout one’s life (Umberson & Karas Montez, 2010)

During the COVID-19 pandemic, the World Health Organization (WHO) recommended precautionary measures, including quarantine, limiting social relations by increasing physical distance, wearing a mask when visiting others, and avoiding overcrowding (Cabello et al.). Due to the importance of this topic, the vulnerability of PLHIV, and the absence of a model examining all the mentioned variables together, especially for this group, the current study aimed to determine relationships among Corona phobia, health anxiety, and social relations in PLHIV via path analysis.

Hence in this study, the following questions are addressed using path analysis which is considered as a causal modelling technique; and can be performed on both cross-sectional and longitudinal data. (Plichta, Kelvin, & Munro)

1-What is the effect of Corona phobia (direct/indirect /total) on the social relation of PLHIV?

2- What is the effect of Health Anxiety (direct/indirect /total) on the social relation of PLHIV?

3- What is the effect of demographic factors such as age, social-economic status, number of partners, and number of children) on the social relation of PLHIV?

2. Methods:

2.1. Study design:

This descriptive-analytical study was conducted in 2021 with PLHIV, who had records at the Behavioral Diseases Center of Tehran University of Medical Sciences (the main referral center for PLHIV).

2.2. Study Population

The sample size was calculated as 300 following Maria Pizzirusso et al. (Pizzirusso et al.), type I and type II error of 0.05 and 0.2, respectively, and the correlation of 0.16 for social relations and anxiety, by using the following formula:

Total sample size = N = [(Zα+Zβ)/C] 2 + 3

C = 0.5 * ln [(1+r)/ (1-r)]

Inclusion Criteria

Iranian men/women with records at the Behavioral Diseases Center, minimum literacy, absence of mental and physical problems (as reported by the patient/registered in their records) that would preclude them from participation, and no history of psychotropic medications.

Exclusion criteria

Returning incomplete questionnaires, migration, and hospitalization due to COVID-19.

2.3. Data collection and definition of terms

The data were collected via four questionnaires: The Fear of COVID-19 Scale (Ahorsu et al.); The Health Anxiety Inventory (Salkovskis & Warwick); social relations questionnaire; the Socioeconomic Status Scale (SES) (Eslami, Mahmoudi, Khabiri, & Najafiyan Razavi); as well as a sociodemographic information checklist.

2.3.1 The the Fear of COVID-19 Scale

Pakpour, Griffiths, et al. developed the Fear of COVID-19 Scale in 2020 with seven items (Ahorsu et al.). The responses range from "strongly disagree" (1) to "strongly agree" (5). The sum of scores of all items yields the total score ranging from 7-35. The original version has a Cronbach's alpha of 0.82, test-retest coefficient of 0.88, and appropriate validity. In Iran, its reliability was confirmed with a Cronbach's alpha of 0.86 (Alizadehfard & Alipour). The scale's reliability was confirmed in the current study with a Cronbach's alpha of 0.84.

2.3.2 The Health Anxiety Inventory

The Health Anxiety Inventory was developed by Salkovskis and Warwick (2002) with 18 items to measure health anxiety. The questions are scored on a four-point Likert scale (never = 0 to often = 3). Salkovskis reported the test-retest reliability of 0.90 and Cronbach's alpha of 0.70-0.82. (Salkovskis & Warwick) The Persian version of the inventory showed a Cronbach's alpha of 0.75, demonstrating optimal validity. (DAVOUDI, NARGESI, & MEHRABIZADEH)

2.3.3. Social relations questionnaire

This questionnaire consists of 11 items scored on a five-point Likert scale from very low to very high (1-5). The score ranges from 11 to 55. Its reliability was confirmed with a Cronbach's alpha of 0.87 (Mousavi, 2013). The current study confirmed its reliability with a Cronbach's alpha of 0.89.

2.3.4. Socioeconomic status scale (SES)

SES consisted of 6 questions, including education of mother and father, income, economic class, and housing status, which are scored based on a Likert scale from 1 to 5, and a total score ranging from 6 to 30. Validity and reliability have been performed in Iran. with a Cronbach's alpha of 0.83 (2013) (Eslami et al.)

2.3.5. Socio-demographic checklist

This scale included questions on the respondents' age, duration of the disease, the number of children, sex partners, education, and having insurance.

2.4. Procedure

The study began after obtaining the required permissions and approval from the Ethics Committee of Alborz University of Medical Sciences (IR.ABZUMS.REC.1400.022). The researchers visiting the Behavioral Diseases Centre identified eligible participants and briefed them about the study's objectives. The eligible participants signed a written informed consent form if they were willing to participate. Due to the COVID-19 pandemic and to adhere to distancing and minimal presence at the Center, the questionnaires were sent to those who had Internet access over the Pars Online platform, and they were requested to fill them out in one week. For those who did not have Internet access, a separate room was allocated for filling out the questionnaires. The respondents could ask their questions regarding questionnaire items and resolve any ambiguities by phone for those who used the Internet and in person for those who filled out the questionnaire in the center.

They were all ensured that their data would remain confidential, that participation was not obligatory, and that they would not be deprived of any services if they did not participate.

2.5. Statistical analysis

This study examined the fitness of a conceptual model for the relationship among fear of COVID-19, health anxiety, and social relations in PLHIV (Figure 1). Path analysis is an extension of conventional regression that shows not only the direct effects but also the indirect effects of each variable on the dependent variables, and the results can be used to provide a rational interpretation of the relationships and correlations observed. Data were analyzed in SPSS-25 and Lisrel-8.8. The results were expressed using Pearson's correlation coefficient for the correlations and in the form of Beta for the path analysis, and the significance level was set at T-value >1.96.

2.6. Research Variables

Socio-demographic characteristics included age, education, child number, SES, partner number, disease duration, and having insurance.

Variables used in the path analysis included Age, education, child number, SES, partner number, health anxiety, corona phobia, and social relation.

3. Results:

The data of 300 PLHIV who had records at the Behavioral Diseases Center of Tehran University of Medical Sciences were investigated. The participants' mean age (39.4 ± 7.5 years), health anxiety score (20.6 ± 7.3), fear of COVID-19 score (22.4 ± 5.3), and social relations score (35.1 ± 3.80 are presented in Table 1.

Based on Pearson's correlation analysis, among variables significantly correlated with social relations, education had the strongest significant positive correlation (r ≈ 0.37) and the number of children displayed the strongest significant negative correlation (r ≈ -0.26) (Table 2).

Based on the path analysis, among variables with significant and causal relationships on the direct path with social relations, socioeconomic status (B = -0.14) had a negative relationship while health anxiety (B = 0.11) had a positive relationship. In other words, with a one-score increase in SES, the social relations score decreased, and with a rise in the health anxiety score, the social relations score increased. On the indirect path, the fear of COVID-19 (B ≈ 0.05) had a significant and positive relationship with social relations; in other words, a rise in fear of COVID-19 score was associated with a rise in social relations score. Level of education was the only variable showing a significant and positive relationship with social relations on both direct and indirect

paths (B = 0.29), meaning that a higher level of education was associated with a higher social relations score (Table 3).

The model's fitness indices demonstrate its goodness of fit, and the reasonably adjusted relationships among the variables (Table 4).

4. Discussion;

This study explored the relationship between fear of COVID-19, health anxiety, and social relations in PLHIV via path analysis. Among variables with significant causal relationships with social relations, SES showed the greatest negative relationship, and health anxiety demonstrated the greatest positive relationship on the direct path. Fear of COVID-19 showed the greatest positive relationship on the indirect path. Level of education was the only variable that had significant and positive relationships with social relations on both direct and indirect paths.

SES had the highest negative relationship with social relations; the poorer the socioeconomic status, the higher the social relations. On the contrary, other studies reported fewer social relations in lower SES groups.(Vonneilich et al.) In another study, patients with HIV who had a lower SES, were poorer, or had lower living standards had fewer social relations than others.(Zhang, Zhang, Aleong, & Fuller-Thomson) This difference in results can be attributed to the pandemic; in our study, those with a higher SES were less in need of being in the society to earn a living or visit healthcare centers, and these factors limit social relations, especially for PLHIV.

Our study revealed that health anxiety had the greatest positive relationship with social relations. We found no similar study on patients with HIV regarding this subject. Nonetheless, a possible cause of this finding could be that patients with greater health anxiety repeatedly visit healthcare centers to check their health status. To ensure the diagnosis, they visit different doctors to make sure they are not infected with COVID-19 (Mohammadi & Shahyad, 2020; Solem et al., 2015), which in turn may increase their social relations. The physical signs and symptoms of health anxiety during the pandemic may resemble the signs and symptoms of COVID-19 itself; in this case, people may mistake these physical changes as symptoms of COVID-19. People with high health anxiety regard any physical change as a sign of a disease, which exacerbates their anxiety and concern, and leads to repeated referrals (Mohammadi & Shahyad).

People with severe immune deficiencies, such as HIV, face numerous side effects. They may be exposed to severe COVID-19 and have a higher mortality risk due to its complications, all of which can cause or exacerbate their stress and concern.(Shiau, Krause, Valera, Swaminathan, & Halkitis)

In the present study, fear of COVID-19 had the strongest positive relationship with social relations through the indirect path. Fear of COVID-19 positively affected health anxiety and thus increased the social relations of the patients. As noted before, PLHIV frequently visit diagnostic and treatment centers due to concerns and fear of COVID-19 complications, which increases their social relations. (Mohammadi & Shahyad, 2020; Solem et al., 2015) COVID-19 research and the media report increased fears of COVID-19. Although fear is a common psychological outcome during the pandemic, it is not limited to morbidity and mortality but may also emerge as social and occupational stress due to the evolving nature of the disease, its prevalence, and its unique risk factors. Corona phobia is a hyper-reactive fear of contracting COVID-19 with three physiologic, cognitive, and behavioural components. Ongoing worry can induce symptoms such as tachycardia, tremor, breathing difficulty, vertigo, a changed appetite, obsession, and affective responses (sadness, guilt, anger). To prevent the consequences, people adopt avoidant behaviours that may disrupt the overall quality of their daily functioning.(Arora, Jha, Alat, & Das) Studies show that the complications and mortality caused by this disease are higher in people with chronic diseases, which induces or exacerbates fear and anxiety in them.(Bakioğlu, Korkmaz, & Ercan, 2021; Mahmoodi & Ghavidel, 2021)

In the current study, level of education was the only variable that had significant and positive relationships with social relations on both direct and indirect paths. Likewise, Nojoumi et al. showed that HIV-positive patients who were educated and employed had a better status than other patients in most quality of life dimensions, especially mental health, social functioning, and environmental dimension. (Nojoomi & Anbari) Educated patients have a better attitude towards the disease and are better adjusted to it due to their better occupational and financial opportunities and high cultural status, which expands their social relations and leads to a better quality of life. Studies show that people with a higher level of education run a longer and healthier life compared to people of the same age but with a lower level of education. (Zajacova & Lawrence) Contrary to our study, a study in China demonstrated no significant relationship between the social relations

of patients with HIV and their level of education. (Liping, Peng, Haijiang, Lahong, & Fan) Cultural differences, economic conditions, and living standards could explain these differences.

5. Conclusion:

This study was conducted during the COVID-19 pandemic when it was necessary to adhere to social distancing and limiting social relations. A better socioeconomic status was correlated with fewer social relations. Moreover, increased fear and health anxiety correlated with a higher score of social relations. Based on their vulnerability, PLHIV require more support and education to adhere to health protocols.

List of abbreviations:

AGE=age of participant

PN= partner number

CN=Child Number

HA=Health anxiety

EDU=Education

CP=Corona phobia

SES=Socioeconomic statues

SR=Social relation

Declarations:

Ethical Approval and consent to participate:

Informed consent was obtained from all the participants entering the study. Relevant guidelines and regulations were observed for all methods. All experimental protocols were approved by the Ethics Committee of Alborz University of Medical Sciences (Abzums.Rec.1399.234). All methods were carried out in accordance with relevant guidelines and regulations

Consent for publication:

All Of Authors have Consent for publication.

Availability of data and material:

The data that support the findings of this study are available from the corresponding author upon reasonable request

Competing interest:

The authors declare that they have no competing interests

Funding:

This research did not receive any specific grant from funding agencies in the public, commercial, or not-for-profit sectors.

Authors' contributions:

F, A. designed and collected the data, performed the statistical analysis and wrote the manuscript. Z, M. supervised the study, contributed to the study design and conducted the analysis, and also helped write the manuscript. M, Q. analyzed the data and helped write the manuscript. M, M and F,M served as the study's scientific advisor, designed and helped write the manuscript. Z, B.J. helped collect the data and write the manuscript. N.GH. and N, MK Helped interpretation and write the manuscript

All author(s) have read and approved the final manuscript.

Acknowledgements;

Authors would like to appreciate the Honorable Vice-Chancellor of Research in Alborz University of Medical Sciences, behavioral disease counselling center of Imam Khomeini Hospital-Tehran due to their scientific support as well as the staff of behavioral disease counselling center of Imam Khomeini hospital and all those who participated in this study.

Additional information

No additional information is available for this paper

Ahorsu, D. K., Lin, C.-Y., Imani, V., Saffari, M., Griffiths, M. D., & Pakpour, A. H. (2020). The fear of COVID-19 scale: development and initial validation. *International journal of mental health and addiction*, 1-9.
The path analysis model in prediction of corona phobia based on .(Alizadehfard, S., & Alipour, A. (2020 intolerance of uncertainty and health anxiety. *Journal of Research in Psychological Health, 14*(1), 16-27.
Asian Journal of Arora, A., Jha, A. K., Alat, P., & Das, S. S. (2020). Understanding coronaphobia. *Psychiatry, 54*, 102384.
Asmundson, G. J., & Taylor, S. (2020). Coronaphobia: Fear and the 2019-nCoV outbreak. *Journal of anxiety disorders, 70*, 102196.
Bakioğlu, F., Korkmaz, O., & Ercan, H. (2021). Fear of COVID-19 and positivity: Mediating role of intolerance of uncertainty, depression, anxiety, and stress. *International journal of mental health and addiction, 19*(6), 2369-2382.
Belir, S., Ansari Shahidi, M., & Mohammadi, S. (2018). Efficacy of Dialectical Behavior Therapy on risky behaviors, depression, anxiety and stress in patients with AIDS. *Health Research Journal, 3*(3), 155-162.
Cabello, A., Zamarro, B., Nistal, S., Victor, V., Hernández, J., Prieto-Pérez, L., . . . Hernández-Segurado, M. HIV: A multicenter case-series study. *International* (2021). COVID-19 in people living with *Journal of Infectious Diseases, 102*, 310-315.
Chenneville, T., Gabbidon, K., Hanson, P., & Holyfield, C. (2020). The impact of COVID-19 on HIV treatment and research: a call to action. *International journal of environmental research and public health, 17*(12), 4548.
Control, C. f. D., & Prevention. (2020). People who are at higher risk for severe illness. 2020. *Accessed May, 12*.
dysfunctional beliefs and DAVOUDI, I., NARGESI, F., & MEHRABIZADEH, H. M. (2012). The Comparison of Personality traits between teachers with and without health anxiety.
Eslami, A., Mahmoudi, A., Khabiri, M., & Najafiyan Razavi, S. M. (2014). The role of socioeconomic public sports. *Applied Research in Sport* conditions in the citizens' motivation for participating in *Management, 2*(3), 89-104.
Fofana, N. K., Latif, F., Sarfraz, S., Bashir, M. F., & Komal, B. (2020). Fear and agony of the pandemic (COVID-19) leading to stress and mental illness: an emerging crisis in the novel coronavirus outbreak. *Psychiatry Research, 291*, 113230.
Liping, M., Peng, X., Haijiang, L., Lahong, J., & Fan, L. (2015). Quality of Life of People Living with HIV/AIDS: A Cross-Sectional Study in Zhejiang Province, China. *PLoS One, 10*(8), e0135705. journal.pone.0135705/10.1371doi:

Mahmoodi, Z., & Ghavidel, N. (2021). Association between Structural Determinants of Health and Posttraumatic Stress Symptoms during the COVID-19 pandemic. *Current Psychiatry Research And Reviews*.
Mertens, G., Gerritsen, L., Duijndam, S., Salemink, E., & Engelhard, I. M. (2020). Fear of the coronavirus (COVID-19): Predictors in an online study conducted in March 2020. *Journal of anxiety disorders, 74*, 102258.
contagious diseases and COVID-19 Mohammadi, M. T., & Shahyad, S. (2020). Health anxiety during viral outbreak: Narrative review. *Journal Mil Med, 22*(6), 623-631.
Nojoomi, M., & Anbari, K. (2008). A comparison of the quality of life in HIV/AIDS patients and control group. *Razi Journal of Medical Sciences, 15*, 169-176.
Pizzirusso, M., Carrion-Park, C., Clark, U. S., Gonzalez, J., Byrd, D., & Morgello, S. (2021). Physical and mental health screening in a New York City HIV cohort during the COVID-19 pandemic: A preliminary report. *Journal of acquired immune deficiency syndromes (1999), 86*(3), e54.
Plichta, S. B., Kelvin, E. A., & Munro, B. H. (2013). *Munro's statistical methods for health care research*: Wolters Kluwer Health/Lippincott Williams & Wilkins.
Porcelli, P. (2020). Fear, anxiety and health-related consequences after the COVID-19 epidemic. *Clinical Neuropsychiatry, 17*(2), 103.
Salkovskis, P. M., & Warwick, H. (2001). Making sense of hypochondriasis: A cognitive theory of health anxiety. In *Health anxiety: Clinical and research perspectives on hypochondriasis and related conditions* (pp. 46-64): Wiley.
Salkovskis, P. M., & Warwick, H. M. (1986). Morbid preoccupations, health anxiety and reassurance: a cognitive-behavioural approach to hypochondriasis. *Behaviour research and therapy, 24*(5), 597-602.
Shiau, S., Krause, K. D., Valera, P., Swaminathan, S., & Halkitis, P. N. (2020). The burden of COVID-19 in people living with HIV: a syndemic perspective. *AIDS and Behavior, 24*(8), 2244-2249.
Symptoms of health .(2015) .Solem, S., Borgejordet, S., Haseth, S., Hansen, B., Håland, Å., & Bailey, R anxiety in obsessive–compulsive disorder: Relationship with treatment outcome and metacognition. *Journal of Obsessive-Compulsive and Related Disorders, 5*, 76-81. doi:https://doi.org/10.1016/j.jocrd.2015.03.002
Tunçel, Ö. K., Pullukçu, H., Erdem, H. A., Kurtaran, B., Taşbakan, S. E., & Taşbakan, M. (2020). COVID-19-related anxiety in people living with HIV: an online cross-sectional study. *Turkish Journal of Medical Sciences, 50*(8), 1792-1800.
Social relationships and health: A flashpoint for health policy. .(2010)Umberson, D., & Karas Montez, J. *Journal of health and social behavior, 51*(1_suppl), S54-S66.
Vonneilich, N., Jöckel, K.-H., Erbel, R., Klein, J., Dragano, N., Siegrist, J., & von dem Knesebeck, O. (2012). The mediating effect of social relationships on the association between socioeconomic status and subjective health – results from the Heinz Nixdorf Recall cohort study. *BMC Public Health, 12*(1), 285. doi:10.1186/1471-2458-12-285
The relationship between education and health: reducing .(018Zajacova, A., & Lawrence, E. M. (2 disparities through a contextual approach. *Annual review of public health, 39*, 273-289.
Zhang, Y., Zhang, X., Aleong, T. H., & Fuller-Thomson, E. (2011). Impact of HIV/AIDS on social relationships in Rural China. *The open AIDS journal, 5*, 67.

Table 1 – the sociodemographic character of Participants (PLWH)

Variables(quantitative)		Mean ± SD	Variables(quantitative)		Mean ± SD
Age		39/4 ± 7/5	Health Anxiety		7.3±20.6
HIV duration		7.88 ± 4.98	Corona Phobia		5.3 ± 22.4
Education		10.07 ± 3.65	Social relation		3.8 ± 35.1
Social-economic status		9.5 ± 2.61	Variables		F (%)
Variables(qualitative)		F (%)	**corona** History	yes	72 (23.8)
				no	230 (76.2)
Insurance status	yes	95 (31.5)	JOB women	Housekeeper	83 (27.5)
	no	207 (68.5)		Worker	84 (27.8)
Number of children	0	116 (38.4)		Employment	23 (7.6)
	1	81 (26.8)		Un employment	47 (15.6)
	2	60 (19.9)		retired	11 (3.6)
	3 and more	45 (14.9)		Self-employment	54 (17.9)

**Table 2. The correlation matrix of personal-social variables, Health anxiety, corona phobia, Social relation in PLWH**									
	Age	Partner number	Child number	HIV duration	Health anxiety	SES	Corona phobia	Social relation	Education
Age	1	-0.152**	0.587**	0.448**	-0.034	0.034	0.124*	-0.235**	-0.356**
Partner number		1	-0.129*	-0.091	-0.069	0.141*	-0.035	0.173**	0.153**
Child number			1	0.289**	0.026	-0.039	0.184**	-0.255**	-0.343
HIV duration				1	-0.081	0.025	0.037	-0.167**	-0.170**
Health anxiety					1	-0.009	0.424**	-0.108*	-0.013
SES						1	0.034	0.192**	0.184**
Corona phobia							1	-0.033	-0.1
Social relation								1	0.365**
Education									1
** : P <0/01 *:P<0/05 , SES: social economic statuse									

**Table 3. The direct and indirect effects among personal-social variables, Health anxiety, corona phobia and Social relation in PLWH**							
	Standard B			Unstandardized β			R^2
	Direct effects	Indirect effects	Total effect	Direct effects	Indirect effects	Total effect	
Age	-0.068	0.01	0.078	-0.034	-0.002	-0.036	
Education	0.276*	0.0145*	0.2905*	0.29*	-0.26*	0.03*	0.49
Child number	-0.118	0.001	-0.117	-0.37	-0.002	-0.372	
SES	-0.140*	-0.001	-0.140*	-0.20*	-0.0015	-0.02	
Partner number	-	0.007	0.007	-	0.015	0.015	

Health Anxiety	0.113*	-	0.113*	0.059*	-	0.059*	
Corona Phobia	-	0.049*	0.049*		0.03*	0.03*	
*Statistically significant, SES: social economic statuse							

Table4: Godnes of fit of Empirical Path Model between health anxiety, corona phobia, and social relations)

Fit Index	X^2	df	X^2/df	CFI	GFI	NFI	RMSEA
Model Index	18.47	8	2.308	0.97	0.98	0.095	0.048
Acceptable range	X2/df < 5			> 0.9	> 0.9	> 0.9	< 0.05

CFI (comparative fit index), GFI (Goodness of fit index), NFI (Bentler-Bonett Normed fit index), RMSEA (root mean squared error of approximation)

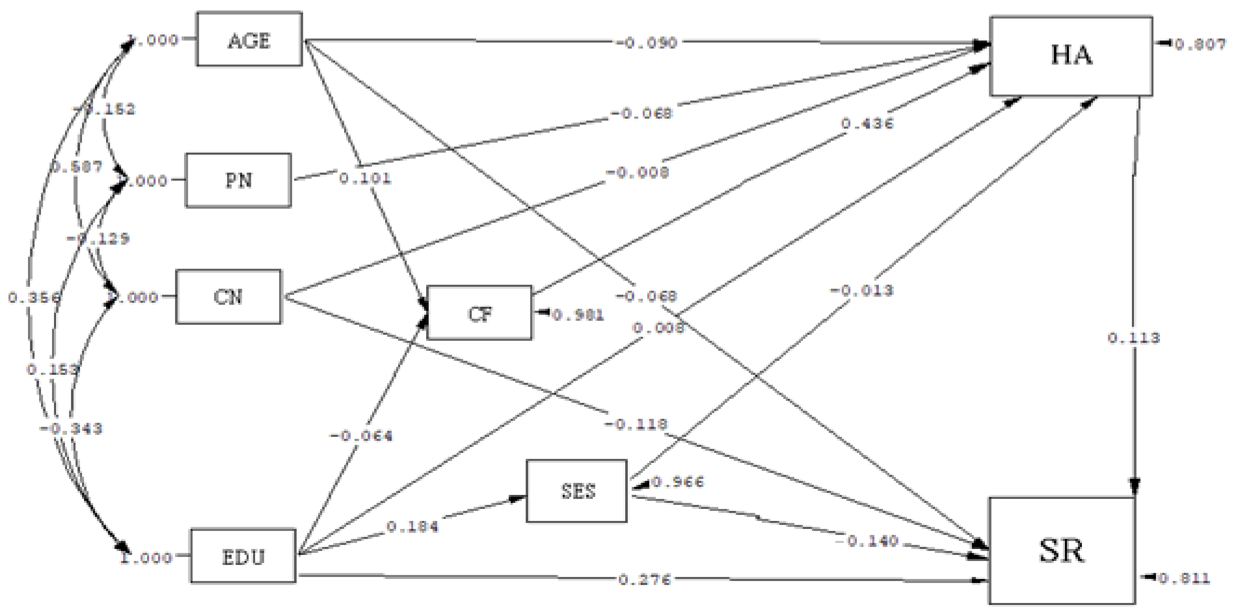

Figure1: Full Empirical Model (Empirical Path Model between health anxiety, corona phobia, and social relations)

AGE=age of the participants, PN= partner number, CN=Child Number, HA=Health anxiety

EDU=Education, CP=Corona phobia, SES=Socioeconomic statues, SR=Social relation

Click here to access/download
Supporting Information
STROBE_checklist_cross-sectional.docx
